# The Uniform System of Accounts

**Published:** 1916-02-26

**Authors:** 


### The Uniform System of Accounts.

*To the Editor of* The Hospital.

Sir,—I do not remember having seen the criticisms of the Uniform System to which you refer in your article of February 12. But there can be no reasonable doubt that in the allocation of expenditure to different headings there is, as you point out, so large a margin of variation that comparison of different hospitals is rendered difficult, if not impossible, in many important particulars. The most flagrant instance of this is, perhaps, in the case of those institutions which have a number of subsidiary concerns (so to speak) which are financially independent but under the same management. For instance, a hospital may have a medical school, a nursing home, and a students' hostel attached; in respect of all of which, if the buildings adjoin, many items of expenditure must be apportioned. The way in which this is done will radically affect the standing of the hospital, but there is at present no guarantee of any uniformity in this vital matter.

For this and similar difficulties you suggest two remedies —a running audit, or, as an alternative, the same auditors for every hospital in London. I venture, with only the most amateur knowledge of auditing, to doubt if either of these suggestions will quite meet the difficulty of the case. It seems doubtful whether any auditors will take the responsibility of deciding that all allocations of expenditure are rightly and properly made; would they, for instance, go behind a minute of the board of management? Again, the same firm of auditors does not mean the same auditor for each hospital, and there may easily be as great a variation between two men employed by the same firm as between different firms.

May I carry your idea one step further and suggest that a single inspector should be appointed by King Edward's Hospital Fund, whose business it would be not to audit the books of each hospital, but to inquire into just those difficult allocations of expenditure which we all know so well—out-patients and in-patients, schools and hostels, and so on—and to see that in every case where no general ruling applicable to all can be made the allocations represent as nearly as possible the work done or the expenditure incurred. Such a post would not be easy to fill; tact, a thorough knowledge of accountancy, a reasonable amount of hospital experience, and a fair endowment of native shrewdness would be essential qualities for any candidate. But such things are to be had if properly paid for, and the right man would undoubtedly exercise a valuable and helpful influence in the world of hospitals.—I am, Sir, yours faithfully,

February 14, 1916. Assistant Secretary.

[It is impossible to make a general rule as to allocation of expenditure between a hospital and subsidiary organisations, as circumstances differ so widely. In London, however, some light is thrown upon what has been done in individual accounts as the total official salaries subject to division must be stated in main and subsidiary accounts as well as the proportion charged to the accounts. A common auditor would see that these divisions are properly made. Auditors cannot always go behind a board minute, but they have strong powers of persuasion. The men employed by a firm of auditors would refer important matters involving principle to their chiefs. Our correspondent's suggested inspector is not practicable for many reasons. He would really not possess the same power as would the auditing firm, or its representative, told off to look after the accounts of a group of hospitals. —Ed. The Hospital.]

